# Iodine Status of Brazilian School-Age Children: A National Cross-Sectional Survey

**DOI:** 10.3390/nu12041077

**Published:** 2020-04-13

**Authors:** Juraci A. Cesar, Iná S. Santos, Robert E. Black, Maria A. D. Chrestani, Fabio A. Duarte, Eduardo A. F. Nilson

**Affiliations:** 1 Postgraduate Program in Public Health, Faculty of Medicine, Universidade Federal do Rio Grande, Rua Visconde de Paranaguá, 102, 4° Piso, Rio Grande 96210.900, RS, Brazil; 2 Postgraduate Program in Epidemiology, Universidade Federal de Pelotas, Rua Marechal Deodoro, 1160-3° Piso, Pelotas 96020.220, RS, Brazil; inasantos@uol.com.br; 3 Department of International Health, Johns Hopkins Bloomberg School of Public Health, 615 North Wolfe Street, Baltimore, MD 21205, USA; RBlack@jhsph.edu; 4 Department of Social Medicine, Faculty of Medicine, Universidade Federal de Pelotas, Av. Duque de Caxias, 250, 3º andar, Pelotas 96030.001, RS, Brazil; machrestani@uol.com.br; 5 Department of Chemistry, Universidade Federal de Santa Maria, Prédio 21, subsolo, Sala 5015—Camobi, Santa Maria 97105.900, RS, Brazil; fabioand@gmail.com; 6 Ministry of Health, Zona Cívico-Administrativa, Brasília 70058900, DF, Brazil; eduardo@saude.gov.br

**Keywords:** iodine, iodine excess, iodine deficiency, iodine intake, urinary iodine concentration, salt iodine concentration, children, schoolchildren, nutrition

## Abstract

Salt iodization is the main public health policy to prevent and control iodine deficiency disorders. The National Salt Iodization Impact Assessment Survey (PNAISAL) was conducted to measure iodine concentration among Brazilian schoolchildren. A survey including 6–14-year-old schoolchildren from public and private schools from all 26 Brazilian states and the Federal District was carried out in the biennia 2008–2009 and 2013–2014. Municipalities, schools, and students were randomly selected. Students were interviewed at school using a standard questionnaire, which included the collection of demographic, educational, weight, height, and 10 mL non-fasting urine collection information. The analyses were weighted according to the population of students per federative unit. The median urinary iodine concentration (MUIC) for the entire sample by region, federative unit per school, and student characteristics, was described from the cutoff points defined by the World Health Organization (severe disability: <20 µg/L, moderate: 20–49 µg/L, mild: 50–99 µg/L, adequate: 100–199 µg/L, more than adequate: 200–299 µg/L, and excessive: >300 µg/L). In total, 18,864 students (95.9% of the total) from 818 schools in 477 municipalities from all federative units were included in this study. Almost 70% were brown skin color, nine-years-old or older, studied in urban schools, and were enrolled in elementary school. The prevalence of overweight/obesity, as measured by body mass index (BMI) for age, was about twice as high compared to nutritional deficits (17.3% versus 9.6%). The MUIC arrived at 276.7 µg/L (25th percentile = 175.5 µg/L and 75th percentile = 399.71 µg/L). In Brazil as a whole, the prevalence of mild, moderate, and severe deficit was 6.9%, 2.6%, and 0.6%, respectively. About one-fifth of the students (20.7%) had adequate iodine concentration, while 24.9% and 44.2% had more than adequate or excessive concentration, respectively. The prevalence of iodine deficits was significantly higher among younger female students from municipal public schools living in rural areas with the lowest BMI. The median urine iodine concentration showed that Brazilian students have an adequate nutritional intake, with a significant proportion of them evidencing overconsumption of this micronutrient.

## 1. Introduction

Iodine, a mineral present in the soil, water, and air, is an essential micronutrient for humans. Intake of iodine depends on the type of diet and the geographic region of residence due to its variable distribution [1]. In the human body, its primary function is to regulate the production of thyroid hormones necessary for cell growth and development, especially nerve cells [2]. Hence, its most considerable importance is in the gestational period and early years of life [2,3].

The iodine deficiency can result in congenital anomalies, abortion, stillbirth, perinatal mortality, impaired cognitive function, delayed physical development, reduced work productivity, hypothyroidism, goiter, hyperthyroidism, among other thyroid disorders [4]. Excessive iodine intakes may precipitate hyperthyroidism, hypothyroidism, goiter, and thyroid autoimmunity, and also result in thyroid disorders among those with preexisting thyroid disease or previously exposed to iodine deficiency [5,6]. 

The method for assessing iodine nutritional status at the population level is the median urinary iodine concentration (MUIC). According to the World Health Organization (WHO), the United Nations Children’s Fund (UNICEF), and the International Council for the Control of Iodine Deficiency Disorders (ICCIDD)—current Iodine Global Network (IGN)—these followings categories from MUIC are defined: severe deficiency: <20 µg/L, moderate: 20–49 µg/L, mild: 50–99 µg/L, adequate: 100–199 µg/L, above adequate: 200–299 µg/L, and excessive: 300 µg/L) [7].

Fortification of salt with iodine has resulted in one of the most cost-effective public health measures because of its impact on child health and the occurrence of iodine deficiency diseases, and the ability to achieve high utilization at very low cost [7,8]. A substantial increase in worldwide iodine consumption has been observed recently [9,10,11]. Between 2007 and 2019, the number of iodine-deficient countries fell from 47 to 25, equivalent to about 600 million more people receiving adequate iodine levels [9,10]. Despite this improvement, about 1.5 billion individuals, of which 0.25 million are schoolchildren, are at risk of iodine deficiency worldwide [10,11].

Brazil has a long history of combating iodine deficiency disorders (IDD). The first Brazilian government measure to combat IDD was established in the mid 20th century when it made salt iodization mandatory in endemic goiter areas. Since then, a number of enhancements were made before the establishment of a national program in 2007 [12,13]. In Appendix A, Panel 1 shows the 1953–2014 actions in Brazil regarding IDD and salt iodization. The first measure dates from 1953, with salt iodization in endemic areas, and the most recent, in 2014, by increasing the amount of iodine to be added per kilogram of salt. In this period, goiter prevalence among school-age children in the country fell from 20.7% in 1955 to 1.4% in 2000 [12,13]. No other study has been conducted since then, but due to the improved social conditions that occurred mainly until 2010, it is possible to suggest that it is very close to zero [14]. Despite these improvements, monitoring iodine concentration is still necessary, because its deficiency is the single most important preventable cause of brain damage [7].

Brazil consists of 26 states plus the Federal District. It has 210 million inhabitants distributed across 8.5 million km^2^ and 5570 municipalities. About 7% of the population over the age of 15 is illiterate, and 1.5% of those of school age are out of school. At least 70% of the population makes use of the public health system at all levels of care. Fertility and under-five-year mortality rates are 1.8 children per women and 14.9 deaths for 1000 live births, respectively. Gross national product per capita/year is U$7900, while the unemployment rate is 11.8% [15].

This study aimed to assess iodine status in a representative sample of schoolchildren aged 6–14 years from all Brazilian states and the Federal District.

## 2. Methods

### 2.1. National Salt Iodization Impact Assessment Survey (PNAISAL)

This study is called the National Salt Iodization Impact Assessment Survey (PNAISAL) and is a population-based survey with representation at country, region, and federative unit levels. It aimed to evaluate the impact of salt iodization consumed in Brazil. PNAISAL is part of the National Program for the Prevention and Control of Iodine Deficiency Disorders that aims to guide, monitor, and evaluate governmental actions and control IDD in Brazil [16].

PNAISAL included students aged 6–14 years enrolled in public and private schools located in urban and rural areas of all Brazilian states and the Federal District in March 2008 to December 2009 and August 2013 to June 2014. These two steps were necessary for financial reasons. The first included students from 18 states and the Federal District, which represented 68% of the total population. The second stage included students from the remaining eight states.

### 2.2. Urinary Iodine Excretion

The amount of iodine was measured in urine collected from the students. MUIC is the most commonly used indicator to assess the nutritional status of iodine at the population level. The WHO, United Nations Children’s Fund (UNICEF), and the International Council for the Control of Iodine Deficiency Disorders/Iodine Global Network (ICCIDD/IGN), define these categories from MUIC: severe deficiency: <20 µg/L, moderate: 20–49 µg/L, mild: 50–99 µg/L, adequate: 100–199 µg/L, above adequate: 200–299 µg/L, and excessive: ≥ 300 µg/L [7].

### 2.3. Sample Size

The 2006 School Census, conducted by the Anísio Teixeira National Institute for Educational Studies and Research (INEP), identified 162,727 schools with 1,237,902 elementary classes and 33,534,561 enrolled students [17]. This represents, on average, 7.6 and 27.1 classes per school and students per class, respectively.

Considering the inclusion of urban and rural schools from each of the 27 federative units, a total of 54 estimated domains would be required. These parameters would result in 171 students per stratum, assuming a priori maximum error of 2.5 points percent for proportions greater than or equal to 0.10, alpha error of 0.05, beta error of 0.10, and a cluster effect of 4.1. This effect was based on the Brazilian Survey on National Endemic Goiter Prevalence, 1994–1995 [18] [Panel 1]. This increased the total number of students per stratum to 701, resulting in 37,854 schoolchildren aged 6–14 years to be identified in the 54 sample strata.

Considering the available resources, the difficulty of a survey of this size, and that urban or rural representativeness per unit is not relevant to managers at the central level, the final sample size was halved (37,854/2 = 18,927). Thus, the data presented here are representative of Brazil as a whole, each geographical region, and each federative unit, but not of urban or rural areas per federative unit.

One class per grade and two students per class in each school were randomly selected to reach the desired 18,927 students. This value was multiplied by the mean number of classes per school in each stratum (7.6, rounded to 8), and could reach 20,121 students. It would be possible to reach the desired sample size with this number, even with some losses.

### 2.4. Collected Information

A single questionnaire seeking information on school characteristics (geographic location, type, and grade offered), the student (demographic, grade level, and mother’s name), and anthropometric measurements (weight and height) was applied. BMI-for-age was calculated as body weight (kg) divided by height (m) squared (kg/m^2^). Z-score is a quantitative measure of the deviation of a specific BMI value from the mean of that population and was calculated with the WHO 2007 growth curves [19].

### 2.5. Operationalization of School-Based Research

The Ministry of Education sent a letter to all the included schools explaining the study and informing them of the PNAISAL team’s visit to that school. This visit was divided into two parts. In the first part, a team member went to the school, confirmed the number of classes, and randomly-selected the classes and two students in each one. Then, the team member would meet with all of them, explain the study, and give them a letter and consent form for their parents to sign. The following week, the team collected the forms, gathered the students to apply the questionnaire, measured weight and height, and collected a 10 mL non-fasting urine sample.

These samples, identified by state, municipality, schools, class, name of the students and their mothers, were stored in cool boxes in this vehicle and later stored in freezers where the team was staying. By the end of the day, the questionnaires were coded by the interviewers and reviewed by the supervisor.

These questionnaires were double-entered, compared, and corrected at the Federal University of Rio Grande (FURG), while the samples were packed in a specific freezer at a temperature of at least −26 °C at the Federal University of Pelotas (UFPel) sample conservation center, both in Southern Brazil. They were later sent for analysis at the two contracted laboratories.

### 2.6. Procedures

Urine was transferred by the interviewer to a 9.5 mL capacity plastic tube with no preservative and conical bottom. These Vacuette-branded materials were manufactured in Austria by Greiner Bio-One. The samples collected in the first phase (13,297 = 67.6% of the total) were analyzed at the Pharmacology Laboratory of the University of São Paulo, Ribeirão Preto, Brazil, through a multiplate persulphate digestion method followed by Sandel–Kolthoff colorimetry, with a detection limit of 0.5 µg L^−1^ [20]. Second phase samples (6383 = 32.4%) were analyzed at the Chemical and Environmental Analysis Laboratory of the Federal University of Santa Maria, Rio Grande do Sul, Brazil. This was because this laboratory is also registered by the Ministry of Health and was the winner of this public tender. In this lab, iodine was determined in an inductively coupled plasma mass spectrometer (NexION 300X, PerkinElmer, Waltham, USA), monitoring the isotope 127I. The accuracy of iodine determination by inductively coupled plasma-mass spectrometry (ICP-MS) was evaluated through the analyte spike at different levels [21].

### 2.7. Statistical Analysis

All data were double entered, compared, and inconsistencies corrected [22]. Initially, the sample distribution was described, and the MUIC was calculated for the entire sample, by region, and federative unit. Since the number of elementary school students in each state ranged from 85,036 in the state of Roraima to 5,978,649 in São Paulo, the data were weighted according to the population of elementary school students in the corresponding state. Then, a bivariate analysis was conducted to verify the concentration of iodine in these students as per their characteristics and the schools where they were enrolled. All tests were two-tailed, and the statistical significance was 95%. The analyses were performed using Stata software [23].

### 2.8. Ethical Aspects

The study protocol was approved by the Research Ethics Committee of the School of Medicine of the Federal University of Pelotas, affiliated to the Brazilian National Research Ethics Commission (CONEP) (OF. No 051/2009). All students handed in the consent form signed by their parents or legal guardians authorizing their participation before their inclusion in the study. During the collection of urine samples, each student received a folder containing information about the properties and functions of iodine, causes, and ways to prevent iodine deficiency, as well as care about the salt storage at home and the importance of its adequate daily consumption.

## 3. Results

This study identified 19,680 students from 818 schools in 477 municipalities in all 26 Brazilian states and the Federal District. From this total, 19,057 (96.8%) students responded to the questionnaire, and 18,978 (96.4%) urine samples were collected, for which 18,864 (95.9%) results were obtained, which is the denominator of this study.

Table 1 shows the distribution of students by region and federative unit. The proportion of students by region ranged from 11.4% (2.157) in the South to 34.5% (6.515) in the Northeast, while by state, it ranged from 2.7% (514) in Rio de Janeiro to 4.3% (806) in Tocantins.

The characteristics of the students are shown in Table 2, with a slight predominance of girls. About 70% of all students were brown skin color, studied in schools located in urban areas, and attended elementary school. Ninety-two percent of them came from public schools, half of which were from municipal schools. The mean age was 10.5 years, with little variation between regions. The prevalence of moderate and severe nutritional deficits using BMI-for-age was about twice as high in less developed regions (Northeast, Midwest, and North) compared to the others (South and Southeast), and this was reversed concerning obesity.

The national MUIC was 276.7 µg/L, ranging from 248.0 µg/L in the South to 298.8 µg/L in the Northeast, while between states, the lowest median was observed in Amazonas (169.3 µg/L), and the largest, in Rio Grande do Norte (361.0 µg/L). Values for the 25th and 75th percentiles are also shown in Table 3. 

According to the classification proposed by the WHO for the assessment of the MUIC, as presented in Table 4, 0.6% of Brazilian schoolchildren have severe deficit (<20 µg/L), 2.6% moderate deficit (20–49 µg/L), 6.9% mild deficit (50–99 µg/L), 20.7% adequate consumption (100–199 µg/L), 24.9% more than adequate consumption (200–299 µg/L), and 44.2% overconsumption (≥300 µg/L). The highest prevalence of severe and moderate deficit (<50 µg/L) was observed in the Northern Region, particularly in the states of Amazonas, Acre, and Tocantins, with 10.5%, 7.1%, and 6.1%, respectively. Regarding the more than adequate and excessive concentration (≥200 µg/L), the highest prevalence was found in the Southeast and Northeast, with at least 72% of the students evidencing this condition. At the state level, the highest rates were observed in Rio Grande do Norte (82.4%) and Espírito Santo (79.3%). 

Table 5 shows the urine iodine concentration by characteristics of the students and the school where they are enrolled. Severe and moderate deficits were more prevalent in girls, brown skin color, younger students (6–8 years) who attended rural and municipal public schools, while the more than adequate or excessive concentration was more commonly observed among older, white boys (12–14 years old) attending urban and private-sector schools (*p* < 0.005).

Finally, Table 5 also shows that the better the BMI status of the student, the higher the urine iodine concentration. The prevalence of iodine deficit (<100 µg/L) was 13.0% among those with <−2 BMI Z-score against 7.1% among those with >+3 BMI Z-score, while the prevalence of excessive urine iodine concentration (>200 µg/L) was inverted, reaching 58.1% and 72.8% in the same groups, respectively (*p* < 0.001).

## 4. Discussion

Having achieved a MUIC of 100 µg/L for at least 90% of schoolchildren and a total median of 276.7 µg/L, it can be concluded that Brazil has largely succeeded in combating iodine deficiency disorders. This success is tempered by the need to address excessive iodine consumption in 44% and remaining deficiency in 10% of the population in a country of continental dimensions.

Data available for 173 countries in the global scorecard of iodine nutrition 2019 show that 25 of them have a MUIC of less than 100 µg/L. However, there is no information on the proportion of the population of these countries that would have reached at least this median consumption considered minimally adequate by the WHO. Regarding the median of 276.7 µg/L, of all countries, only 16 of them have a value higher than that observed in this study [10]. A recent meta-analysis with studies from different locations has shown a significant reduction in the prevalence of iodine deficiency and goiter (grade 1–2), which suggests that iodine intake has been increasing globally [24].

Of the 10% with iodine deficiency, two-thirds were classified as mild, which is still of concern because it can reduce intellectual function and motor skills and increase thyroid volume and the risk of goiter in the future [25,26]. Because it affects a more significant number of individuals and interrupts the progression to more severe conditions, combating these types of deficiencies is also essential, hence the need to universalize the supply of iodized salt and monitor the quality of salt consumed and the amount excreted in the urine. Unlike what is commonly believed, these measures are also necessary for high-income countries such as Italy, Norway, and the United Kingdom, which are iodine-deficient [10]. In the UK, a recent study carried out with schoolgirls aged 14–15 years attending secondary school in nine centers, the prevalence of mild, moderate, and severe deficiency were present in 51%, 16%, and 1%, respectively [27].

Although widespread throughout Brazil, the iodine deficiency is most prevalent in states of the Legal Amazon, which includes all states in the Northern region and some of the Midwest and Northeast regions. In some states of this area, such as Amazonas, Acre, Maranhão, and Piauí, the prevalence of iodine deficiency is at least 70% higher than the national mean. This lower consumption of iodine may be due to the difficulty of accessing urban areas to buy salt, due to the long distance and poor road quality. Because of this and the fact that cattle breeding is widespread in this region, families may be consuming the same salt used for these animals, which is generally not iodized. Still, regarding the higher deficiency prevalence in the Legal Amazon, another possibility is that, in border municipalities, households may be consuming salt from neighboring countries, which may not be iodized. These two aspects must be considered when addressing this deficiency in the region. Finally, children with worse nutritional status, which frequently occur in this region, may be affected by sporadic goiter. Low levels of retinol-binding protein are found in this situation, which leads to defective synthesis of retinol phosphate-mannose, which affects the thyroglobulin iodination process, resulting in non-utilization of available iodine [28].

Excess iodine affected 44% of the population studied and was more prevalent in the Southeast, the richest, and Northeast, where salt consumed in the country is produced. It was more prevalent among males, older students living in urban areas, enrolled in private schools, and with higher BMI-for-age, as expected. This excess results from higher food consumption and the household’s place of residence. The higher the intake of mainly processed foods, the greater the possibility of ingesting salt and, therefore, iodine. 

The place of residence is also about access to iodized salt. Families living in urban areas, besides better purchasing power, are more likely to acquire salt and various other processed products containing large amounts of salt and, thus, more iodine. These products are widely consumed by them, which elevates the MUIC. In Brazil, at least two national studies on food consumption conducted in the same period of PNAISAL were found. Both, one with household sampling [29] and the other with school sampling [30], concluded that, in this age group, the most prevalent items were sweets, milk drink, cookies, chips, sausages, and soft drinks, all rich in salt. These products are highly available and cheaper in urban areas, nonexistent in rural areas and, if available, expensive in municipalities distant from large urban centers. Specific policies discouraging this practice and promoting the consumption of healthy foods must be established at the national level.

At least two limitations may have affected the results of this study. The first concerns data collection in two moments (2008–2009 and 2013–2014), with a five-year interval. This is unlikely to have significantly changed the MUIC because the most significant changes in access to consumer goods and social indicators occurred in Brazil, mainly until 2010. Little changed, or even deteriorated, since then, such as the unemployment rate and household income [31].

Participation of private schools was voluntary, while it was compulsory for public schools. This meant that 23 private schools, out of 72 initially planned, did not participate in the study. Concerning these schools, we were unable to know the number of classes, grades, or students enrolled, which prevented their inclusion in the calculations. However, considering that these children generally have better socioeconomic status than those in public schools, it can be assumed that, if included in the study, the MUIC would increase. We can assume from these limitations that these results are slightly overestimated concerning the prevalence of deficiency and underestimated regarding excess intake. This would not greatly change the results, whether due to the lack of improved indicators in the period (2008–2009 and 2013–2014), or the non-participation of private school students, which would have little weight in the total sample.

However, it is vital to highlight the challenges of conducting a representative study, with a sample size not previously observed for primary data, in a country of this dimension and numerous logistical difficulties such as Brazil. Despite the difficulty in reaching many rural locations, especially in the Legal Amazon and Semiarid Northeastern region, whether due to long distances, inadequate means of transportation, or poor road conditions, the losses in this study were low (4.1%) and the proportion of samples analyzed was successfully high (99.4%). This reveals the extreme care in the selection of individuals and the conservation of samples to ensure its internal validity. Finally, from the results of this study, the amount of iodine per kilogram of salt in Brazil decreased from 20–60 mg to 15–45 mg [32] [Panel 1].

Our data show that Brazil has done its homework by achieving adequate levels of iodine consumption for almost the entire population. This makes it one of the first countries to virtually eliminate iodine deficiency diseases, far ahead of more economically and socially equitable developing countries or even high-income countries. 

The Brazilian government should continue to ensure iodization of salt and its distribution nationwide [Panel 1]. Regarding the deficiency, it is necessary to investigate the reasons why the prevalence in some states of the Legal Amazon is about twice as high compared to the rest of the country. Regarding excessive iodine intake, it seems appropriate to re-evaluate the amount of salt allowed in processed foods, besides establishing a nationwide campaign to deter the consumption of salt and junk food. Otherwise, this excessive intake can facilitate the occurrence of chronic lymphocytic thyroiditis [33].

It would also be appropriate to establish a sentinel surveillance system for iodine status of schoolchildren, prioritizing states with more deficiency, i.e., the Amazon region, and the states with the high median urinary iodine concentration, to monitor iodine consumption and consider changes in the salt iodization program in a more timely way.

## Figures and Tables

**Table 1:** Distribution of schoolchildren aged 6 to 14 by region and state, Brazil, 2008–2014.

Region	State	n	Percent
**South**	Paraná	740	3.9
	Rio Grande do Sul	759	4.0
	Santa Catarina	658	3.5
	Total	2157	11.4
**Southeast**	Espírito Santo	694	3.7
	Minas Gerais	686	3.6
	Rio de Janeiro	514	2.7
	São Paulo	643	3.4
	Total	2537	13.5
**Northeast**	Alagoas	696	3.7
	Bahia	648	3.4
	Ceará	708	3.8
	Maranhão	780	4.1
	Paraíba	800	4.2
	Pernambuco	694	3.7
	Piauí	789	4.2
	Rio Grande do Norte	637	3.4
	Sergipe	763	4.0
	Total	6515	34.5
**Midwest**	Federal District	721	3.8
	Goiás	672	3.6
	Mato Grosso	735	3.9
	Mato Grosso do Sul	744	3.9
	Total	2872	15.2
**North**	Acre	705	3.7
	Amapá	634	3.4
	Amazonas	635	3.4
	Pará	755	4.0
	Rondônia	656	3.5
	Roraima	592	3.1
	Tocantins	806	4.3
	Total	4783	25.4
Total		18,864	100.0

**Table 2:** Characteristics of schoolchildren aged 6 to 14 years by region, 2008–2014.

Variables	Region					Total
	South	Southeast	Northeast	Midwest	North	
Sex						*p* = 0.007
Female	48.0%	51.2%	51.8%	51.4%	52.8%	51.5%
Male	52.0%	48.8%	48.2%	48.6%	47.2%	48.5%
Skin color						*p* < 0.001
White	66.0%	34.1%	17.5%	27.0%	14.9%	26.1%
Brown	31.2%	57.7%	76.9%	68.5%	82.1%	69.1%
Black	2.8%	8.2%	5.5%	4.5%	3.0%	4.8%
Age (in years)						*p* < 0.001
6 to 8	32.0%	28.3%	26.2%	28.2%	23.0%	26.6%
9 to 11	32.2%	32.3%	30.0%	31.9%	31.6%	31.3%
12 to 14	35.8%	39.4%	43.8%	39.9%	45.3%	42.1%
Mean (Standard deviation)	10.1 (2.6)	10.4 (2.5)	10.6 (2.6)	10.4 (2.6)	10.7 (2.5)	10.5 (2.6)
School location area						*p* < 0.001
Urban	75.1%	79.5%	62.3%	74.2%	62.4%	67.9%
Rural	24.9%	20.5%	37.7%	25.8%	37.6%	32.1%
Type of School						*p* < 0.001
Public, state	51.4%	44.5%	24.8%	47.2%	56.5%	41.9%
Public, municipal	45.1%	44.3%	66.4%	40.0%	38.4%	49.9%
Private	3.5%	11.2%	8.8%	12.8%	5.1%	8.2%
School grade						*p* < 0.001
Elementary (only)	61.2%	59.5%	80.8%	65.6%	60.6%	68.3%
Elementary/Middle	38.8%	40.5%	19.2%	34.4%	39.4%	31.7%
BMI-for-age (Z-score)						*p* < 0.001
<−3	0.1%	0.2%	0.4%	0.4%	0.4%	0.3%
>−3 and <−2	0.4%	0.9%	1.4%	1.3%	1.1%	1.1%
>−2 and <+1	4.5%	7.4%	10.3%	8.2%	7.5%	8.2%
>+1 and <+2	70.1%	71.7%	73.4%	72.2%	74.8%	73.0%
>+2 and <+3)	17.4%	13.3%	10.7%	13.4%	12.4%	12.7%
>+3	7.4%	6.4%	3.7%	4.4%	3.7%	4.6%
Total	100%	100%	100%	100%	100%	100%
(n)	(2157)	(2537)	(6515)	(2872)	(4783)	(18,864)

**Table 3:** Standard deviation and median urine iodine concentration for schoolchildren aged 6 to 14 years by region and states, Brazil, 2008–2014.

Region	State	N	Urine Iodine Concentration (µg/L)			
			Mean (Standard Deviation)	Percentiles		
				25th	50th (Median)	75th
**South**	Paraná	740	276.3 (156.8)	173.7	254.9	343.0
	Rio Grande do Sul	759	283.2 (176.5)	174.5	252.6	350.7
	Santa Catarina	658	256.5 (157.2)	145.5	226.7	336.5
	Total	2157	272.7 (164.4)	166.2	248.0	343.2
**Southeast**	Espirito Santo	694	356.3 (188.6)	222.6	325.4	461.3
	Minas Gerais	686	306.0 (169.0)	184.1	292.5	399.9
	Rio de Janeiro	514	295.7 (145.9)	184.5	277.2	376.9
	São Paulo	643	302.5 (163.3)	191.0	283.2	391.6
	Total	2537	316.3 (170.7)	195.0	295.2	405.2
**Northeast**	Alagoas	696	379.7 (201.1)	234.9	345.8	502.2
	Bahia	648	345.5 (219.3)	189.2	306.0	441.2
	Ceará	708	332.7 (215.6)	183.5	296.9	440.5
	Maranhão	780	268.5 (187.7)	123.2	234.7	375.5
	Paraíba	800	364.7 (206.7)	221.2	319.0	477.0
	Pernambuco	694	330.2 (188.4)	208.8	300.9	411.0
	Piauí	789	249.9 (167.0)	132.4	218.7	329.5
	Rio Grande do Norte	637	420.8 (260.8)	237.0	361.0	556.4
	Sergipe	763	343.2 (180.9)	216.8	318.6	448.4
	Total	6515	334.8 (209.0)	188.8	298.8	441.4
**Midwest**	Federal District	721	278.0 (148.7)	177.6	260.4	354.9
	Goiás	672	272.8 (157.3)	159.6	261.4	364.7
	Mato Grosso	735	291.7 (172.3)	170.2	265.6	381.3
	Mato Grosso do Sul	744	308.4 (172.0)	184.7	284.0	391.1
	Total	2872	288.2 (163.6)	172.8	267.5	374.4
**North**	Acre	705	262.3 (182.2)	134.2	229.8	338.4
	Amapá	634	371.2 (261.1)	205.0	308.2	464.0
	Amazonas	635	241.6 (185.5)	105.4	197.6	324.0
	Pará	755	311.1 (202.0)	175.3	280.9	389.3
	Rondônia	656	271.6 (191.9)	145.3	232.4	350.1
	Roraima	592	322.7 (168.0)	195.4	296.8	420.2
	Tocantins	806	313.0 (224.5)	158.7	272.9	410.8
	Total	4783	299.0 (208.5)	158.0	261.0	391.0
Total		18,864	309.0 (194.1)	175.5	276.7	399.7

**Table 4:** Iodine concentration in µg/L by region and State, Brazil, 2008–2014.

Region	State	n	Urine Iodine Concentration (µg/L)					
			Severe Deficit (<20)	Moderate Deficit (20–49)	Mild Deficit (50–99)	Adequate (100–199)	More than Adequate (200–299)	Excessive (≥300) *p* < 0.001
**South**	Rio Grande do Sul	759	0.1%	2.0%	6.5%	26.5%	29.2%	35.7%
	Santa Catarina	658	0.3%	3.6%	9.7%	25.5%	29.5%	31.3%
	Paraná	740	0.5%	1.5%	7.4%	23.0%	30.9%	36.6%
	Total	2157	0.3%	2.3%	7.8%	25.0%	29.9%	34.7%
**Southeast**	São Paulo	643	0.8%	2.2%	6.2%	18.2%	27.2%	45.4%
	Rio de Janeiro	514	0.2%	1.0%	4.3%	23.5%	27.6%	43.4%
	Minas Gerais	686	0.1%	2.4%	7.7%	19.1%	23.0%	47.5%
	Espirito Santo	694	0.1%	0.9%	3.9%	15.8%	22.2%	57.1%
	Total	2537	0.3%	1.7%	5.6%	18.9%	24.8%	48.8%
**Northeast**	Sergipe	763	0.0%	1.3%	5.4%	14.5%	24.1%	54.6%
	Rio Grande do Norte	637	0.0%	1.7%	3.6%	12.2%	20.7%	61.7%
	Piauí	789	0.8%	5.7%	10.3%	28.1%	25.2%	29.9%
	Pernambuco	694	0.0%	0.6%	5.0%	17.9%	26.4%	50.1%
	Paraíba	800	0.0%	0.6%	5.1%	13.7%	25.2%	55.2%
	Maranhão	780	2.7%	5.9%	11.1%	22.6%	19.6%	38.1%
	Ceará	708	0.4%	1.8%	7.2%	19.3%	21.9%	49.3%
	Bahia	648	0.0%	1.2%	6.9%	19.0%	21.6%	51.2%
	Alagoas	696	0.1%	0.7%	3.2%	14.4%	21.1%	60.5%
	Total	6515	0.5%	2.3%	6.5%	18.1%	22.9%	49.6%
**Midwest**	Mato Grosso do Sul	744	0.5%	1.3%	5.1%	21.4%	26.9%	44.8%
	Mato Grosso	735	0.5%	2.0%	7.3%	22.6%	25.0%	42.4%
	Goiás	672	1.0%	3.3%	8.8%	21.6%	24.8%	40.5%
	Distrito Federal	721	0.4%	2.1%	6.1%	22.7%	30.9%	37.7%
	Total	2872	0.6%	2.2%	6.8%	22.1%	26.9%	41.4%
**North**	Tocantins	806	1.4%	4.7%	7.4%	21.7%	21.0%	43.8%
	Amapá	635	0.3%	1.7%	4.6%	17.2%	24.4%	51.7%
	Roraima	592	0.0%	1.9%	3.4%	20.9%	24.2%	49.7%
	Rondônia	656	0.9%	3.0%	9.8%	6.7%	26.2%	33.4%
	Pará	755	0.5%	2.8%	6.6%	20.1%	25.2%	44.8%
	Amazonas	634	2.5%	8.0%	13.2%	26.9%	20.3%	29.0%
	Acre	705	1.4%	5.7%	9.6%	24.0%	27.7%	31.6%
	Total	4783	1.0%	4.0%	7.8%	22.5%	24.1%	40.5%
Total		18,864	0.6%	2.6%	6.9%	20.7%	24.9%	44.2%

**Table 5:** Urine iodine concentration (µg/L) according to characteristics of schoolchildren aged 6 to 14 years, Brazil, 2008–2014.

Variables	Urine Iodine Concentration (µg/L)					
	Severe Deficit (<20)	Moderate Deficit (20–49)	Mild Deficit (50–99)	Adequate (100–199)	More than Adequate (200–299)	Excessive (≥300)
Sex						*p* < 0.001
Male	0.5%	2.3%	5.8%	19.5%	24.1%	47.9%
Female	0.7%	2.9%	8.0%	21.9%	25.7%	40.8%
Skin color						*p* = 0.020
White	0.3%	2.6%	6.9%	20.8%	25.9%	43.5%
Brown	0.7%	2.6%	6.9%	20.6%	24.4%	44.7%
Black	0.7%	2.0%	7.3%	21.4%	26.8%	41.9%
Age (in years)						*p* < 0.001
6 to 8	0.7%	3.2%	8.1%	22.5%	25.0%	40.4%
9 to 11	0.7%	2.9%	7.2%	21.3%	25.4%	42.5%
12 to 14	0.4%	2.0%	6.0%	19.1%	24.5%	48.0%
School location area						*p* < 0.001
Urban	0.4%	1.9%	5.6%	19.3%	25.3%	47.4%
Rural	1.1%	4.1%	9.6%	23.7%	24.0%	37.5%
Type of School						*p* < 0.001
Public, state	0.4%	2.3%	5.8%	20.5%	25.5%	45.5%
Public, municipal	0.9%	3.0%	8.2%	21.3%	24.1%	42.5%
Private	0.0%	2.3%	4.7%	18.1%	26.1%	48.8%
School grade						*p* = 0.281
Elementary (only)	0.7%	2.6%	7.1%	20.6%	24.8%	44.2%
Elementary/Middle	0.5%	2.6%	6.5%	21.0%	25.1%	44.3%
BMI-for-age (Z-score)						*p* < 0.001
<−2	0.4%	3.6%	9.0%	28.9%	19.5%	38.6%
>−2 and <+1	0.8%	3.3%	6.7%	21.2%	25.7%	42.3%
>+1 and <+2	0.6%	2.7%	7.3%	20.7%	24.5%	44.1%
>+2 and <+3	0.5%	2.0%	5.3%	19.3%	25.8%	47.0%
>+3	0.3%	1.8%	4.9%	20.1%	28.8%	44.0%
Total	0.6%	2.6%	6.9%	20.7%	24.9%	44.2%
(*n* = 18,864)	113	493	1306	3908	4696	8348

## Appendix A

**Panel 1—Timeline for Combating Iodine Deficiency Diseases in Brazil.**	
**1953**	- Enactment of law that mandated the iodination of salt for human consumption in endemic goiter areas.
**1955**	- First national survey conducted with 86,217 students, which found a goiter prevalence of 20.7% in the country.
**1956**	- Delimitation of endemic goiter areas, determination of salt iodination in endemic areas, and transfer of iodate imports to the Ministry of Health.
**1974**	- Determination of the obligation of salt iodization by salt producers, at 10–30 mg/kg of salt, with the supervision of states and municipalities.
**1974–1976**	- Second national survey with 421,752 schoolchildren, which identified goiter prevalence of 14.1%, and found about 15 million with goiter.
**1975**	- Establishment of the salt quality and identification standard, creation of the Endemic Goiter Fighting Program, transfer of salt iodization to the Ministry of Health.
**1984**	- Establishment of sentinel municipalities in several states to periodically monitor endemic goiter levels.
**1990**	- Third Survey in sentinel municipalities by the National Institute of Food and Nutrition, with 16,803 students, which found a prevalence of goiter ranging from 16.4% to 39.9%.
**1994**	- Establishment of the National Iodine Deficiency Disorders Control Program and increase of the amount of iodine per kilogram of salt to 40–60 mg of iodine.
**1994–1995**	- Fourth goiter prevalence survey conducted with 178,774 schoolchildren from 428 sentinel and borderline municipalities. Prevalence of goiter found in mild (19.4%), moderate (3.5%), and severe forms (0.9%).
**1999**	- Salt iodation returns to the private sector, the amount of iodine per kilogram of salt increases to 40–100 mg, and the Interagency Commission for the Control of Iodine Deficiency Disorders is established in the Ministry of Health.
**2000**	- Implementation of the Thyromobil Project of the International Council for Control of Iodine Deficiency Disorders (ICCIDD)—current Iodine Global Network (IGN)—in 17 sentinel municipalities in six Brazilian states. Among the 1977 schoolchildren evaluated, the median urinary iodine was 360 μg/L, and the prevalence of goiter was 1.4%. The mean concentration of iodine in salt consumed at home was 48.3 ± 28.9 ppm mg kg−1.
**2000**	- Implementation of the Pilot Project in the state of Tocantins to monitor the consumption of iodized salt through the action of the State Health Community.
**2003**	- The amount of iodine per kg of salt is reduced to 20–60 mg.
**2005**	- Establishment of a new commission to monitor all actions by the federal government to prevent and control IDD in Brazil.
**2007**	- Establishment of the National Program for the Prevention and Control of IDD (Pro-Iodine) aiming at the elimination of IDD by monitoring the impact of salt iodation on the population’s health, updating the legal parameters of salt iodine levels intended for human consumption, and continuing implementation of information, education, communication, and social mobilization strategies.
**2008**	- Implementation of the National Survey for the Evaluation of the Impact of Salt Iodization (PNAISAL), with 18,864 students from 1865 schools in 479 Brazilian municipalities.
**2014**	- From the PNAISAL results, the amount of iodine per kilogram of salt is redefined at 15–45 mg.
